# *In vitro* and *In silico* studies of interactions of cathinone with human recombinant cytochrome P450 CYP(1A2), CYP2A6, CYP2B6, CYP2C8, CYP2C19, CYP2E1, CYP2J2, and CYP3A5

**DOI:** 10.1016/j.toxrep.2022.03.040

**Published:** 2022-03-30

**Authors:** Sharoen Yu Ming Lim, Jason Siau Ee Loo, Mustafa Alshagga, Mohammed Abdullah Alshawsh, Chin Eng Ong, Yan Pan

**Affiliations:** a Division of Biomedical Science, School of Pharmacy, University of Nottingham Malaysia, 43500 Semenyih, Malaysia; b Centre for Drug Discovery and Molecular Pharmacology, Faculty of Health and Medical Sciences, Taylor’s University, 47500 Selangor, Malaysia; c Department of Pharmacology, Faculty of Medicine Universiti Malaya 50603 Kuala Lumpur, Malaysia; d School of Pharmacy, International Medical University, Bukit Jalil Wilayah Persekutuan, 57000 Kuala Lumpur, Malaysia

**Keywords:** Cathinone, CYP450, in vitro, *in silico*, Docking, Herb-drug interaction, Khat, *Catha edulis Forsk*

## Abstract

Cathinone is the psychostimulatory major active ingredient of khat (*Catha edulis Forsk*) and are often co-abused with alcohols and polydrugs. With the increased consumption of khat and cathinones on a global scale, efforts should be channelled into understanding and minimising the excruciating effects of possible khat-drug interactions. This study aimed to determine the in vitro inhibitory effects of cathinone on CYP1A2, CYP2A6, CYP2B6, CYP2C8, CYP2C19, CYP2E1, CYP2J2 and CYP3A5 and the *in silico* identification of their type of interactions and residues involved. The activities of CYP1A2, CYP2A6, CYP2B6, CYP2C8, CYP2C19, CYP2E1, CYP2J2 and CYP3A5 were examined by fluorescence based assays using recombinant cDNA-expressed human CYPs in Vivid® P450 screening kits. Cathinone reversibly inhibited CYP1A2, CYP2A6 and CYP3A5 via competitive, uncompetitive and noncompetitive modes with inhibition constant (K_i_) values of 57.12, 13.75 and 23.57 µM respectively. Cathinone showed negligible inhibitory effects on CYP2B6, CYP2C8, CYP2C19, CYP2E1 and CYP2J2. Cathinone showed negligible time dependent inhibition on all 8 CYPs. Docking studies was performed on cathinone with CYP1A2, CYP2A6 and CYP3A5 following their inhibition in vitro. Cathinone is bound to a few key amino acid residues in the active sites while π-π interactions are formed in aromatic clusters of CYP1A2 and CYP3A5. These findings offer valuable reference for the use of cathinones and khat when combined with therapeutic drugs that are metabolised by CYP enzymes especially patients on medications metabolised by CYP1A2, CYP2A6 and CYP3A5.

## Highlights

• Active ingredient of khat plant, cathinone, inhibits human drug metabolising enzymes CYP450.
• Herb-drug interactions often occur when users take herbs and medications concurrently.
• Docking studies identify amino acid residues and interactions binding cathinone in CYPs.
• Avoid taking khat/cathinone with drugs especially metabolised by CYP1A2, CYP2A6 and CYP3A5.

## Graphical Abstract

**Figure:**
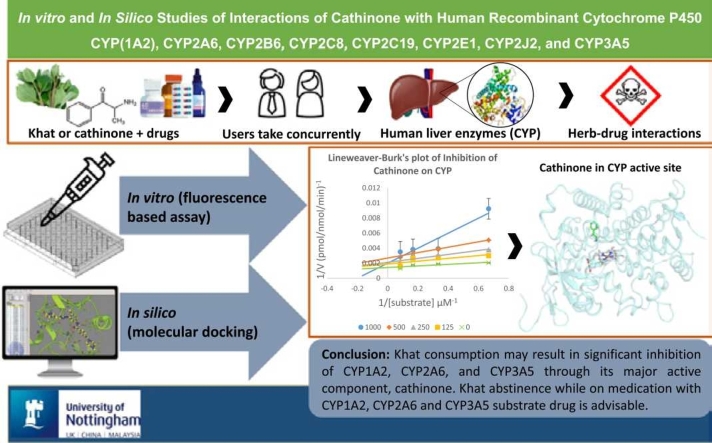

## 1 Introduction

Cathinone or (S-(-)-α-aminopropiophenone) is the major psychostimulant active ingredient found in the leaves of khat (*Catha edulis Forsk*) plant that exists in a few spectra of colours ranging from green to red [24]. Khat chewing and khat sessions are deeply rooted in the cultural traditions among people in the khat-belt countries including Saudi Arabia and East Africa [8]. Large commercial scale of khat cultivation are situated in the Harar province of Ethiopia, Jebel Sabr Mountains of Yemen, Nyambene area of Meru, Kenya, and to a lesser extent in South Africa, Uganda, Tanzania, Zimbabwe, Rwanda and Indonesia (Nilesh B. [51]). Besides that, khat is also available and advertised on the Internet (Nilesh B. [51]). In the UK before the prohibition of khat use, most supplies of khat was imported to Heathrow Airport from khat-belt countries before transported to warehouse in Southall [9] and could be found in East London, specifically in Tower Hamlets [37]. Based on the 34th meeting of WHO Expert Committee on Drug Dependence (ECDD) in 2006, khat is not under international control but cathinone and cathine was included in Schedule I and III respectively, of the UN Convention on Psychotropic Substances in 1988 [70]. Khat is controlled in countries including Belgium, Denmark, Germany, Greece, France, Ireland, Italy, Latvia, Lithuania, Poland, Slovenia, United States, Finland, Sweden, Norway and Switzerland (https://www.emcdda.europa.eu/publications/drug-profiles/khat_en) whereas in Australia, khat import up to maximum of 5 kg for personal use is allowed for licensed individuals only [70].

Ethiopia as the leading khat producer in East African region showed a prevalence of khat chewing of 58% from 1700 individuals in Hossana, of which 68.4% were daily khat users while 31.5% are occasional users [58]. Besides, khat chewing is also popular among medical students with prevalence of 21.5% [2]. Khat use was found to be significantly associated to religion, gender, age, and habit of family and friends [58]. Studies also found that lifetime prevalence of khat use was the highest in Yemen, followed by Saudi Arabia and Ethiopia which were 43.27%, 37.32% and 24.82% respectively [10]. Khat’s global distribution was aided by air transport making khat available in European, UK and US markets [23], [4]. The availability of khat was also thought to be enhanced by development of capsular form of cathinone, known as hagigat [23]. Most khat users in the Western world are immigrants from khat-belt countries [23]. Evidences regarding uptake by the major population in the non-khat-belt countries are lacking due to the unfamiliar mode of administration and the availability of more active stimulants, amphetamine and cocaine [23]. As compared to khat-belt countries, data on prevalence of khat chewing in non-khat-belt countries is rather limited. Back in the 2000 s, khat users in the UK obtained khat from the *mafresh*, a meeting place to purchase and chew khat [3]. About 60% of khat users chewed 1 or 2 bundles of khat in an average khat session that lasted 6 h, with each bundle weighing approximately 250 g of khat, costing about £ 3–5 [3]. A study on male Yemeni residents in Sheffield and Birmingham, UK, found that 83% of the users started khat chewing in Yemen at an average of 17 years, with 73% of them chewed khat more than two days per week [36].

Besides cathinone, khat contains other less psychoactive components including cathine S,S-(+)-norpseudoephedrine, R,S-(-)-norephedrine, catheduline and cathidine [24], [27]. After the young leaves matures, cathinone is metabolised into cathine and norephedrine [6]. Older khat leaves are lacking in cathinone but they have less active cathine and norephedrine [38]. Dried khat leaves stored for months led to dramatic drop of cathinone amount from 0.115% to 0.158% to 0.021–0.023% [53]. Krizevski et al. proposed that cathinone acts as the biosynthetic precursor of cathine and norephedrine in khat leaves [38]. However, red khat contains on average a higher cathinone percentage out of the total phenylpropylamino alkaloids fraction as compared to green khat [38].

**Fig. 1:**
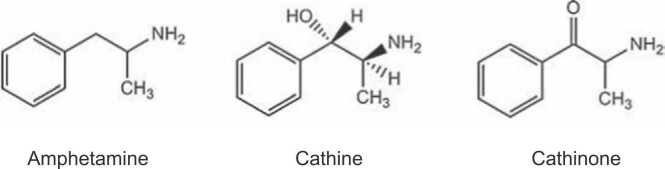
Closely related chemical structures of amphetamine, cathine and cathinone.

Cathinone and cathine are structurally related amphetamines (Fig. 1) [67]. The chemical structure of cathinone is epitomised by a characteristic β-keto group on the side chain of the corresponding phenethylamine [18]. The only structural difference between amphetamine and cathinone is the carbonyl group situated in the α-position of cathinone’s side chain adjacent to aromatic ring [44]. Cathinones are classified as controlled substances [18].

The psychostimulatory effect of khat is primarily due to cathinone because the lipophilicity of cathinone is greater than cathine which favour its central nervous system (CNS) penetration, while peripheral effects are due equally to actions of cathinone and cathine (N. B. [50]). Cathinones exert highly similar effects on the CNS by increasing catecholamine concentrations in the inter-synapse region and the potency of this psychostimulant is much stronger than amphetamine but less than methamphetamine [18], [44]. The effects of cathinone use on users are considered subjective and intoxicated individuals are said to experience euphoria, psychosis, self-injurious behaviour, hypertension, myocardial infarction, seizures and headaches for instances [44], [66]. Khat-drug interactions was reported by Abebe [1] stating khat interactions with various types of drugs metabolised by cytochrome P450 (CYP)2D6 [1]. Referring to these widely documented health threats of khat, cathinone abuse plus their co-administration with conventional drugs, research in this area is very much needed to shed light to the understandings of the possible occurrence of khat-drug interactions. Moreover, the mechanism of action of khat compounds with CYPs in all aspects including *in silico*, in vitro and in vivo are very much warranted for an overall picture.

CYP isoenzymes are a family of haem-containing enzymes embedded in the lipid bilayer of endoplasmic reticulum of liver cells, responsible for drug, steroids and carcinogen metabolism [14]. In humans, CYP families 1–3 are involved in biotransformation of foreign substances and 70 – 80% of all clinical drug metabolism [74]. Approximately 90% of clinical drug oxidation is catalysed by CYP1A2, CYP2C9, CYP2C19, CYP2D6, CYP2E1, CYP3A4 and CYP3A5 [14]. CYP3A4, CYP2C9, CYP2C8 and CYP1A2 are more highly expressed in the liver as compared to CYP2A6, CYP2D6, CYP2B6, CYP2C19 and CYP3A5 whereas CYP2J2, CYP1A1 and CYP1B1 are chiefly expressed extra-hepatically [74]. Herbal medicines and phytochemical mixtures were found to be capable of inducing or inhibiting the CYP enzymes [17]. Our previous study reported in vitro enzyme activities of CYP2D6, CYP2C9, CYP3A4 was significantly inhibited by khat ethanol extract (KEE) [43]. Based on our findings, CYP inhibition by khat extracts could be caused by the many alkaloids present in khat as aforementioned, and could also be a combination of the major constituents in khat, namely cathinone and cathine, or with a mix of other constituents. A recent study using LC-MS/MS method to explore the effect of khat on pharmacokinetics of an antiplatelet drug, clopidogrel, in rats also substantiated our previous findings that herb-drug interactions should be taken into consideration during prescribing to ensure an optimal dosage of drugs such as clopidogrel [7].

Despite our previous findings demonstrating that cathinone showed negligible in vitro inhibitory effects on CYP2C9, CYP2D6, and CYP3A4 [43], as the major compound present in khat, cathinone may exert its inhibitory effects on other CYPs isoform. Moreover, research exploring the in vitro effects and protein-ligand interactions of cathinone on major human drug metabolising CYPs namely CYP1A2, CYP2A6, CYP2B6, CYP2C8, CYP2C19, CYP2E1, CYP2J2, and CYP3A5 are lacking. This study is prudent to project into and visualise the possible mechanism of cathinone’s interactions with CYP active sites and the surrounding amino acid residues. *In silico* prediction of CYP-ligand interactions may shed light to the understanding the dynamics of CYP ligand binding recognition and affinity, prediction of inhibitors, their potency and the possible metabolites formed [59]. Therefore, continuing our previous study, this current study aimed to explore the inhibitory effects of CYP1A2, CYP2A6, CYP2B6, CYP2C8, CYP2C19, CYP2E1, CYP2J2, and CYP3A5 in vitro using cDNA-expressed recombinant human CYPs, in addition to computational molecular docking studies to understand how cathinone interact with these CYPs.

## 2 Materials and methods

### 2.1 Chemicals and reagents

Cathinone or S(-)-Cathinone.HCl ((S)− 2-Amino-1-phenyl-1-propanone.hydrochloride) was procured from Lipomed AG (Arlesheim, Switzerland). Acetonitrile was purchased from Fisher Scientific (Loughborough, Leicestershire, UK) while tris-base powder was obtained from Amresco® LLC (Solon, Ohio, USA). The human recombinant Vivid® kits namely CYP1A2, CYP2A6, CYP2B6, CYP2C8, CYP2C19, CYP2E1, CYP2J2 and CYP3A5 was procured from Life Technologies™ (Carlbad, CA, USA). Costar black 96-well plates were obtained from Thermo Fisher Scientific (Pittsburg, PA, USA).

### 2.2 Standard and time curves

Standard curves equations were derived by plotting fluorescence readings (RFU) produced by i) blue (3-cyano-7-hydroxycoumarin) standard with Buffer I, II and III, ii) cyan (7-hydroxy-4-trifluoromethylcoumarin) standard with Buffer II, and iii) green (fluorescein) standard against a range of concentrations (0, 3.905, 7.81, 15.6, 31.25, 62.5, 125, 250, 500 nM) of the respective standard to obtain the standard equations and R^2^ values [42]. The standard curve equations were employed in subsequent assays to quantify the fluorescent metabolites formed. A range of incubation time (0, 5, 10, 20, 30, 60, 120 min) was carried out to plot the time curves for CYP1A2, CYP2A6, CYP2B6, CYP2C8, CYP2C19, CYP2E1, CYP2J2 and CYP3A5. Linear ranges of the CYPs were chosen: 60 min for CYP1A2, CYP2A6, CYP2B6, CYP2C8, CYP2C19, CYP2E1, CYP2J2 and 120 min for CYP3A5 [42].

### 2.3 Determination of CYPs activity using vivid P450 assay kits

The inhibitory effects of cathinone on the catalytic activities of cDNA-expressed human CYP450 enzymes was determined using Vivid® CYP450 Screening Kits for all CYP isoforms according to manufacturer’s instructions [41]. The Vivid® CYP450 Screening Kits employed in this study were; Vivid® EOMCC CYP1A2 Blue, Vivid® CC CYP2A6 Blue, Vivid® BOMCC CYP2B6 Blue, Vivid® DBOMF CYP2C8 Green, Vivid® EOMCC CYP2C19 Blue, Vivid® EOMCC CYP2E1 Blue, Vivid® MOBFC CYP2J2 Cyan and Vivid® BOMCC CYP3A5 Blue.

Briefly, the assays were performed in black 96-well plates in kinetic assay mode. In each well, 40 µL of respective reaction buffers (Buffer I: CYP1A2, CYP2B6, CYP3A5; Buffer II: CYP2A6, CYP2C8, CYP2C19, CYP2J2; Buffer III: CYP2E1) was incubated with 50 µL of master premix (including CYP450 BACULOSOMES® Plus, human CYP reductase, potassium phosphate buffer, NADPH regeneration system containing glucose-6-phosphate buffer (333 mM), 0.3 U/ml glucose-6-phosphate dehydrogenase in 100 mM potassium phosphate at pH8.0) at room temperature for 30 min. The reaction was initiated by addition of 10 µL per well of a mixture of respective substrates and NADP^+^. Therefore, the total volume for incubation per well was 100 µL in the 96-well plate.

Following the 30 min pre-incubation, the CYP enzyme specific substrates [Vivid® BOMCC (7-benzyloxymethyloxy-3-cyanocoumarin) for CYP2B6 and CYP3A5; Vivid® CC (3-cyanocoumarin) for CYP2A6; Vivid® DBOMF (dibenzylmethylfluorescein) for CYP2C8; Vivid® EOMCC (ethoxymethyloxy-3-cyanocoumarin) for CYP1A2, CYP2C19 and CYP2E1; Vivid® MOBFC (7-p-methoxy-benzyloxy-4-trifluorocoumarin) for CYP2J2] and 0.03 mM NADP^+^ were added, and the mixture was shaken in room temperature for 60 or 120 min based on the CYP isoform. The reaction was stopped by addition of 50 µL of 0.5 M tris-base solution and the enzyme activity was evaluated by measuring the fluorescence using Varioskan® Fluorescence Spectrophotometer (Thermo Fisher Scientific®, Waltham, MA, USA) at excitation/emission wavelengths of 415/460 nm (blue – CYP1A2, CYP2A6, CYP2B6, CYP2C19, CYP2E1, CYP3A5), 415/520 nm (cyan – CYP2J2) and 490/520 nm (green – CYP2C8).

### 2.4 Reversible inhibition

Cathinone stock of 2.5 mM was prepared by dissolving cathinone in water. The assay conditions were as described above with exception that the 40 µL of buffer were substituted with 40 µL of cathinone stock at concentrations ranging from 0 to 1000 µM following two times serial dilution. The percent control activity (%) of each cathinone concentration was derived by dividing the remaining enzyme activity at each cathinone concentration, with the solvent control well (without cathinone but replaced by water) and multiplying the value with 100%. The resulting percent control activity (%) were plotted against different concentrations of cathinone to attain the half maximal inhibitory concentrations (IC_50_) curve. K_i_ values and inhibition modes were further assessed for cathinone on CYP1A2, CYP2A6 and CYP3A5 based on their respective IC_50_ values of less than 100 µM. Different concentrations of cathinone (0, 31.25, 62.5, 125, 250, 500, 1000 µM) was incubated with Vivid® Fluorogenic Probe Substrates EOMCC (1.5, 3, 6, 12 µM) for CYP1A2, CC (5, 10, 20, 40 µM) for CYP2A6 and BOMCC (5, 10, 20, 40 µM) for CYP3A5.

### 2.5 Determination of time-dependent inhibition (TDI)

The procedure followed was similar to that used for screening of CYP reversible inhibition as mentioned above but with slight modifications. The master premix was added with NADP^+^ to produce NADPH before the pre-incubation. The mixture was incubated for 30 min with shaking under similar range of concentrations of cathinone as mentioned above for IC_50_ determination. Substrates were added after 30 mins pre-incubation and fluorescence readings are taken after 60 min for CYP1A2, CYP2A6, CYP2B6, CYP2C8, CYP2C19, CYP2E1, CYP2J2 and 120 min for CYP3A5 incubation. The IC_50_ shift was derived by calculating ratios of IC_50_ obtained from pre-incubation with and without NADPH. An IC_50_ shift ratio of more than 2 signifies irreversible time-dependent inhibition [13].

### 2.6 Molecular docking

Interactions of cathinone with human CYP1A2, CYP2A6 and CYP3A5 were analyzed by molecular docking studies. X-ray crystal structures of human CYP CYP1A2 (PDB code: 2HI4), CYP2A6 (PDB code: 2FDV) and CYP3A5 (PDB code: 6MJM) were retrieved from Protein Data Bank [12]. The crystal structure of human CYP2J2 could not be solved and therefore, Rabbit CYP2B4 (PDB code: 1SUO) was selected for docking in this study [56]. The rabbit CYP2B4 has high sequence similarity of 87% to human CYP2J2, having complete sequence and high resolution of 1.9 Å [56].

Open Babel GUI was employed to generate initial 3D coordinates of cathinone (O′Boyle 2011). AutoDock 4.2 [45] was used to carry out molecular docking while protein and ligand structures was processed using AutoDock Tools 1.5.6. Grid maps were calculated using AutoGrid, with the grid box centred around the co-crystallised ligand and covering the haem-containing active site. The grid size for specifying the search space was set at 60 × 60×60 Å (for CYP1A2, CYP3A5) and 70 × 70×70 Å (for CYP2A6) centred on the macromolecule with a default grid point spacing of 0.375 Å. Docking simulations were performed using the Lamarckian genetic algorithm. Default values were used for other parameters. During the docking simulation, the enzyme structure was kept rigid, while the ligand was left fully flexible. The top-ranked binding modes and protein-ligand interactions were visualized with PyMOL Molecular Graphics system (Schrödinger, LLC, New York, NY, USA).

### 2.7 Data analysis

The IC_50_ values of reversible and irreversible inhibition were determined by nonlinear regression analysis using GraphPad Prism 9 for Windows (GraphPad Software, San Diego, CA). The secondary plots of cathinone concentrations against slopes of Lineweaver-Burk plots were plotted to determine the K_i_ values. The mode of inhibition was determined graphically from the Lineweaver-Burk plot using Microsoft Excel. All assays were carried out in triplicate and stated as mean ± SD.

## 3 Results

### 3.1 Standard curves and time curves

Standard equations and R^2^ values used in this study have been obtained from earlier studies [42]. Time curves incubation time was also determined previously: 60 min for CYP1A2, CYP2A6, CYP2B6, CYP2C8, CYP2C19, CYP2E1, CYP2J2 and 120 min for CYP3A5 [42].

### 3.2 Inhibition of cathinone on CYPs

**Fig. 2:**
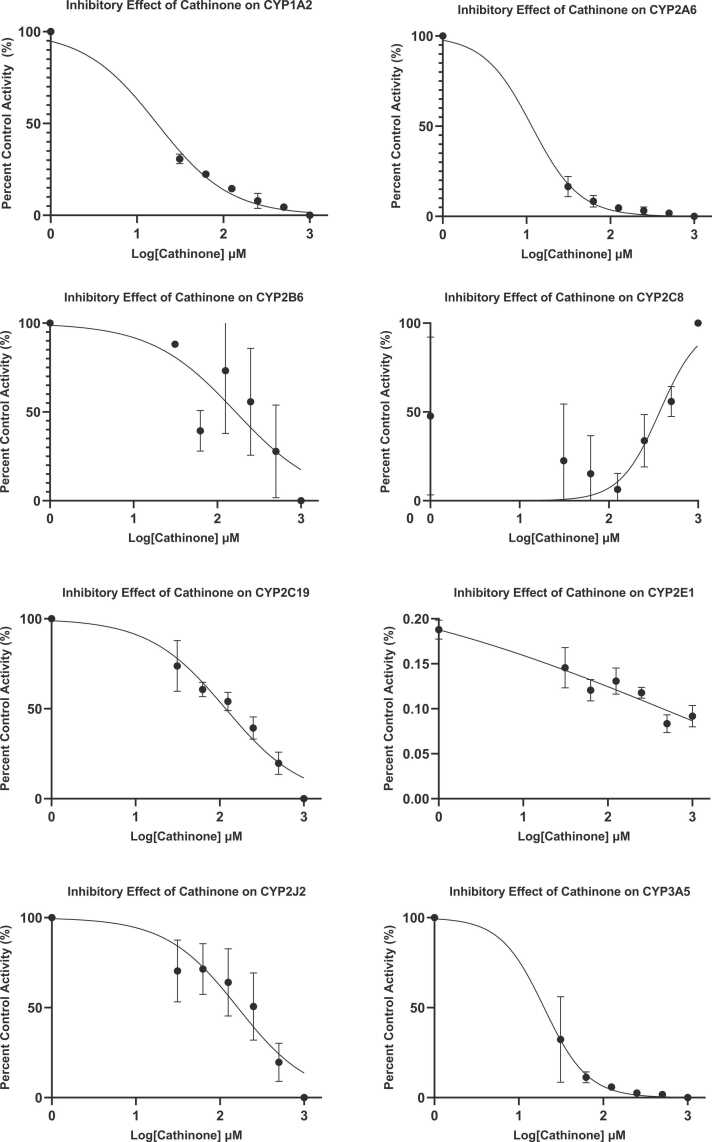
Inhibitory effects of cathinone on (A) CYP1A2, (B) CYP2A6, (C) CYP2B6, (D) CYP2C8, (E) CYP2C19, (F) CYP2E1, (G) CYP2J2 and (H) CYP3A5. IC_50_ values were determined by nonlinear regression analysis using GraphPad Prism version 9 for Windows (GraphPad Software, La Jolla California, USA). Each point represents mean ± SD (n = 3). *CYP* Cytochrome P450, *IC*_*50*_ 50% inhibitory concentration.

Cathinone inhibited CYP1A2, CYP2A6 and CYP3A5 with IC_50_ (mean ± SD) values of 17.01 (25.71 ± 34.43), 11.53 (19.22 ± 36.04) and 19.88 (21.95 ± 36.15) µM respectively as shown in nonlinear graphs (Fig. 2). Cathinone showed no significant inhibition on CYP2B6, CYP2C8, CYP2C19, CYP2E1 and CYP2J2 with IC_50_ values of 165.1 (54.89 ± 35.27), n.d., 117.5 (49.66 ± 33.48), 568.5 (40.15 ± 33.42), 162.7 (53.73 ± 33.92) µM respectively, which were more than 100 µM.

### 3.3 Time dependent inhibition (TDI)

Table 1**.** shows the determined IC_50_ without and with NADPH in pre-incubation and IC_50_ shift for CYP1A2, CYP2A6, CYP2B6, CYP2C8, CYP2C19, CYP2E1, CYP2J2 and CYP3A5. An IC_50_ shift of > 2 will be considered as significant TDI potency [13]. Cathinone showed no significant TDI potency on all 8 CYPs (refer Supplementary Information Fig. 1).

**Table 1:** IC_50_ without/with NADPH, in pre-incubation and IC_50_ shift of CYP1A2, CYP2A6, CYP2B6, CYP2C8, CYP2C19, CYP2E1, CYP2J2 and CYP3A5 with cathinone.

CYP isoform	IC_50_ without NADPH (µM)	IC_50_ with NADPH (µM)	IC_50_ shift
CYP1A2	17.01	75.36	0.23
CYP2A6	11.53	13.71	0.84
CYP2B6	165.1	110.4	1.50
CYP2C8	n/a	n/a	n/a
CYP2C19	117.5	219.7	0.53
CYP2E1	568.5	71.26	0.73
CYP2J2	162.7	193.6	0.84
CYP3A5	19.88	11.05	1.80

*n/a = not applicable

### 3.4 K_i_ analysis and mode of inhibition

**Fig. 3:**
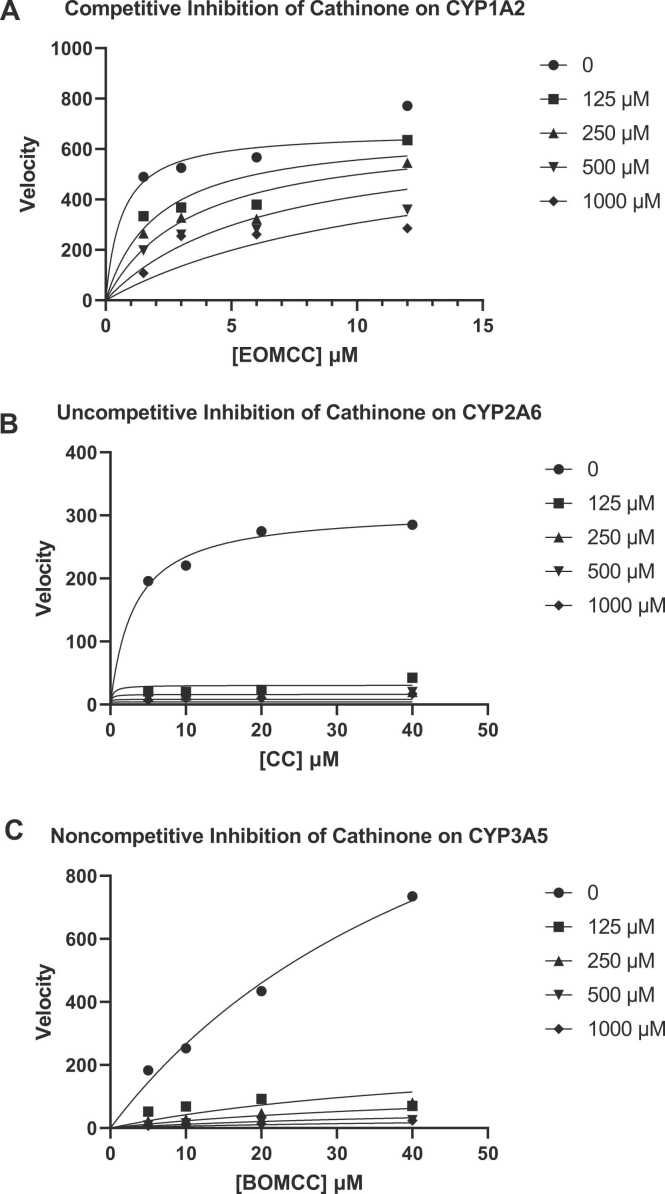
The nonlinear regression – enzyme kinetics graphs were plotted by using velocity against individual CYP’s substrate concentration. The mode of inhibition was (A) competitive inhibition of CYP1A2 by Cathinone; (B) uncompetitive inhibition of CYP2A6 by Cathinone and (C) noncompetitive inhibition of CYP3A5 by Cathinone. The substrate concentration used were; EOMCC (1.5, 3, 6, 12 µM) for CYP1A2, CC (5, 10, 20, 40 µM) for CYP2A6 and BOMCC (5, 10, 20, 40 µM) for CYP3A5. Each data point are triplicates that was represented by mean ± SD (n = 3).

The K_i_ values were obtained from secondary plots of each CYP isoforms. Cathinone inhibited CYP1A2, CYP2A6 and CYP3A5 via competitive mode with K_i_ of 57.12 µM, un-competitive mode with K_i_ of 13.75 µM, and noncompetitive mode with K_i_ of 23.57 µM as shown in the nonlinear regression – enzyme kinetics graphs (Fig. 3). The Lineweaver-Burk’s and secondary plots of CYP1A2, CYP2A6 and CYP3A5 are showing comparable mode of inhibitions (refer Supplementary Information Fig. 2).

### 3.5 Molecular docking

**Fig. 4:**
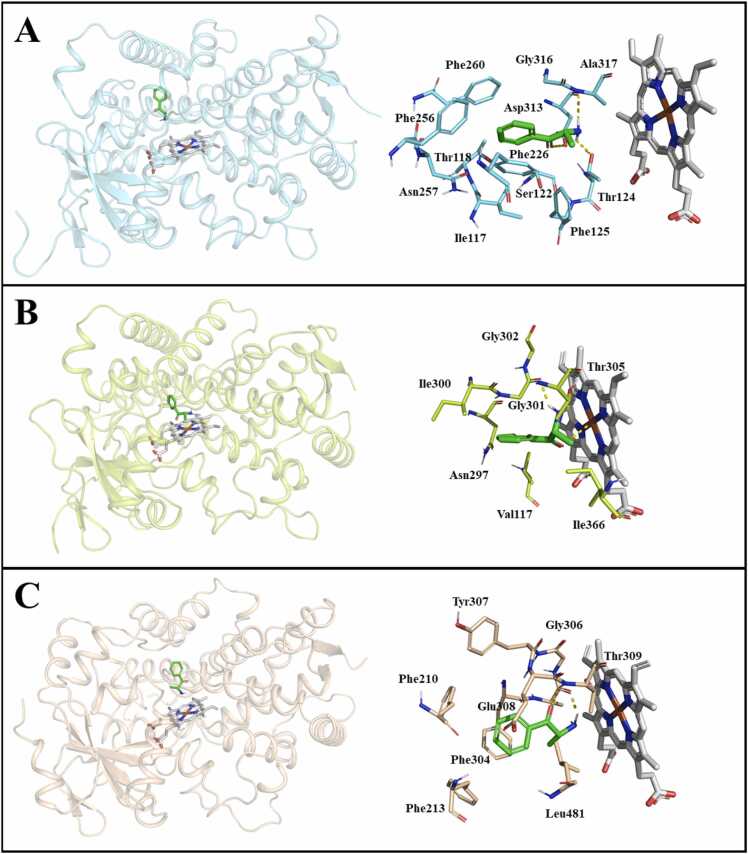
Molecular docking demonstrating binding modes and key interactions of cathinone (green) relative to the haem group (white) in the active sites of (A) CYP1A2 (PDB 2HI4); (B) CYP2A6 (PDB 2FDV); and (C) CYP3A5 (PDB 6MJM). Hydrogen bonds are displayed as yellow dashed lines. Non-polar hydrogens have been removed for visual clarity. (For interpretation of the references to colour in this figure legend, the reader is referred to the web version of this article.)

Cathinone was docked to the active sites of human CYP1A2, CYP2A6 and CYP3A5 to explore its binding mode and interactions. The binding energies, amino acid residues and type of interactions between cathinone and CYP1A2, CYP2A6, CYP3A5 were as shown in Fig. 4 and Table 2.

**Table 2:** CYPs binding energies and interacting residues.

CYP isoforms	Binding energy (kcal mol^−1^)	Amino acid residues	Interaction
CYP1A2	– 6.85	Ile117, Thr118, Ser122, Phe125, Phe256, Gly316	Hydrophobic
		Thr124, Asp313, Ala317	Hydrogen bond
		Phe226, Phe260	π-stacking
CYP2A6	– 7.78	Val117, Asn297, Ile300, Gly302, Thr305, Ile366	Hydrophobic
		Gly301, Thr305	Hydrogen bond
CYP3A5	– 6.53	Phe210, Phe213, Phe304, Gly306, Glu308, Leu481	Hydrophobic
		Glu308, Thr309	Hydrogen bond
		Phe304	π-stacking

## 4 Discussion

Cathinone has been used as antidepressants and anorectic drugs in the 1960 s but have evolved into a popular drug of abuse [55]. Similar to khat, cathinone is commonly taken together via “mix and matching” with psychoactive and non-psychoactive substances, with or without alcohol that may cause complications [31]. Polysubstance use involving cathinone (e.g. mephedrone) accompanied with alcohol [49] and co-abuse of controlled drugs such as heroin, cocaine, cannabis, ketamine and MDMA are popular among users [26]. Moreover, the concurrent polysubstance use among khat chewers are alcohols, cigarettes [47], cardiovascular drugs [7], cannabis, and benzodiazepine tablets, just to name a few [46]. Khat chewing reduces the bioavailability of co-administered antibiotics and antimalarial drugs, counteract with antihypertensive, antiarrhythmic and local anaesthetics but enhanced the effects of monoamine oxidase inhibitors and amphetamine like drugs [1].

Based on our previous findings, cathinone showed negligible inhibitory effects on human CYP2D6, CYP2C9 and CYP3A4 but KEE (khat ethanol extract) inhibited CYP2C9 via non-competitive or mixed mode with K_i_ of 14.7 μg/ml, CYP2D6 through competitive or mixed mode with K_i_ of 17.6 μg/ml, CYP3A4 by mixed inhibition mode with K_i_ of 12.1 μg/ml in vitro [43]. KEE also inhibited CYP2A6, CYP2B6, CYP2C8, CYP2C19, CYP2E1, CYP2J2 and CYP3A5 in vitro with K_i_ values of 20.9, 85, 4.8, 18.3, 59.3, 3, and 21.7 μg/Ml [42]. Continuing the previous study as aforementioned, this study aimed to determine the in vitro inhibitory effects of cathinone on CYP1A2, CYP2A6, CYP2B6, CYP2C8, CYP2C19, CYP2E1, CYP2J2, CYP3A4 and CYP3A5 enzyme activities and *in silico* studies were carried out for CYPs demonstrating significant inhibition to explore the interactions of cathinone within their active sites. Most studies focus on khat’s effect on CYPs using human subjects or rodents. In this study, cathinone reversibly inhibited CYP1A2 via competitive mode of inhibition with IC_50_ (mean ± SD) and K_i_ value of 17.01 (25.71 ± 34.43) and 57.12 µM; CYP2A6 via uncompetitive mode of inhibition with IC_50_ and K_i_ value of 11.53 (19.22 ± 36.04) and 13.75 µM; and CYP3A5 via noncompetitive mode of inhibition with IC_50_ and K_i_ values of 19.88 (21.95 ± 36.15) and 23.57 µM respectively (Figs. 2 and 3). During competitive inhibition, cathinone competes with substrate for binding to the CYPs active site [52]. When cathinone occupies the active site, it forms an enzyme-cathinone complex, inhibition CYP’s reaction until cathinone dissociates [52]. Competitive inhibitors are usually substrate analogs which have similar structures to the substrate but are unreactive [52]. In noncompetitive inhibition, cathinone acting as the inhibitor binds to the allosteric site but not the active site of the CYP enzymes or enzyme-substrate complex [39] while in uncompetitive inhibition, cathinone binds to the enzyme-substrate complex [48]. CYP inhibitors can either bind to the active site or allosteric site and if the inhibitors binds to both sites, this phenomenon is called mixed inhibition [21]. The inhibitor (in this case cathinone) can either be substrates or non-substrates of CYP as non-substrates will usually bind to the allosteric sites [21]. Therefore, cathinone in this study could be an inhibitor, substrate or non-substrate based on the noncompetitive and uncompetitive inhibition observed. Cathinone showed negligible time dependent or mechanism-based inhibition of CYP1A2, CYP2A6, CYP2B6, CYP2C8, CYP2C19, CYP2E1, CYP2J2, and CYP3A5 (Table 1).

To further understand interactions between cathinone and the major human drug metabolising CYPs, molecular docking analysis was performed. The binding energies, amino acid residues and type of interactions between cathinone and CYPs were as described in Table 2. Based on the conformations in Fig. 4, cathinone’s orientation within the CYP active site was in close proximity to haem group of CYP1A2, CYP2A6 and CYP3A5. The binding pocket of the CYP1A2 is relatively planar and small, with an estimated volume of 375 Å^3^ and its binding cavity fits closely with planar compounds [59]. Based on the docking analysis, residues namely Ile117, Thr118, Phe125, Phe256, Asp313, Ala317, Phe226 and Phe260 were found to be important residues for the binding of cathinone on CYP1A2 (Fig. 4A), consistent with prior research suggesting that Ile117, Thr118, Phe125, Phe226, Phe256, Phe260, Asp313, played important roles in ligand recognition by CYP1A2 [62]. The conserved aromatic residues that contributed to CYP1A2 rigidity or protein structural flexibility and channel conformation allowing ingress of planar polyaromatic substrates (T. [76]) was also found in our cathinone-CYP1A2 model. Moreover, π-π stacking interactions is the most essential determinants in CYP1A2 inhibition potency [62] which is agreement to the docking outcome in this study where π-π stacking interactions was formed between cathinone and the aromatic clusters namely Phe226 and Phe260 (Table 2). Similar π stacking interactions with Phe226 residue located at the top of the CYP1A2 active site with naringenin, the flavone found in grapefruit and CYP1A2 inhibitor, was also observed [61]. The current in vitro study found that cathinone inhibited CYP1A2 in a competitive mode which is warranted and applicable to the docking predictions aforementioned.

The binding pocket of CYP2A6 on the other hand is compact, with a volume of 260 Å^3^, catalyzes metabolism of small planar substrates besides containing three phenylalanines enabling π-π interactions with aromatic compounds and asparagine forming hydrogen bonding [59]. Docking results predicted that Val117, Asn297, Ile300, Thr305 and Ile366 were critical residues for the binding of cathinone to CYP2A6 (Fig. 4B). These interactions might be responsible for inhibitory activity of cathinone against CYP2A6. Asn297 is important determinant of inhibition potency and was considered as the lone available H-bonding site in CYP2A active sites [22], [57]. Moreover, the conserved amino acid Asn297 played significant steric roles [22] which was also found in our cathinone-CYP2A6 model. Asn97 acts as hydrogen bond donor that orients coumarin (1,2-benzopyrone), a prototypic substrate of CYP2A6 within the hydrophobic active site [73]. In this study, we observed slight difference where cathinone is orientated in a way that its polar interactions are with the Fe2 + ion in haem, Gly301 and Thr305. The observed hydrogen bonding between cathinone with Thr305 may also increase the inhibition potency. The tight interaction with haem group could potentially contribute to slightly more potent inhibition of CYP2A6 as seen in the binding energy values of – 7.78 kcal mol^−1^. Studies have illustrated that nicotine (phytotoxin present in many plants) is primarily metabolised by CYP2A6 and the hydrogen bonding by residue Thr305 is crucial for effective binding of pyridine scaffold of nicotine within CYP2A6 [29].

The binding pocket of CYP3A4 and CYP3A5 is well- known to be relatively large and flexible, both enzymes have been recognised with more than two different ligand-binding sites [19]. Residues Phe210, Phe213, Phe304, Glu308, Leu481 participated in hydrophobic interactions, formed hydrogen bonds with Glu308, Thr309 and π-stacking interactions by Phe304 with cathinone in CYP3A5 (Fig. 4C). Residues near the Phe304, i.e., Phe210, Phe213, Glu308 and Leu481, formed hydrophobic interactions to stabilise the phenyl group of cathinone in the binding model. Hydrogen bond interactions appeared to be an important determinant for ligand binding in CYP3A5. Similar to the findings, hydrogen bonding was also observed between lapatinib (oral tyrosine kinase inhibitor used for breast cancer and tumours that may cause hepatotoxicity) and Thr309 in CYP3A5 [15]. Hydrogen bond and hydrophobic interactions of the active site residues played distinguished roles in stabilising substrate/ligand binding [71]. These results provided valuable information on structure-activity relationships between cathinone and CYPs. However, there are several limitation of docking analysis including the flexibility of CYP enzymes [59]. The in vitro results showed uncompetitive as well as noncompetitive inhibition and therefore, the docking results are only applicable to represent interactions of competitive inhibition that occurred within the active sites, as the allosteric site of these CYPs were still actively being investigated. A recent computational modelling study investigated the conserved allosteric site on drug metabolising CYPs in prokaryotes and human CYPs, denoted as hotspot 1 (H1), located among helices C, E and H [28]. CYP1A2, CYP2A6, CYP2B6, CYP2C8, CYP2C19, CYP2D6 were capable to bind small molecules or organic solvents at their H1 sites except CYP2C9, CYP2E1 and CYP3A4 [28]. Further studies are recommended to investigate whether cathinone inhibits drug metabolising CYPs and subsequently be metabolised by CYPs or otherwise. Besides that, the type of interactions and crucial amino acid residues involved within the allosteric site H1 of human drug metabolising CYPs are also worthy to be further explored.

From our study, cathinone, the major active ingredient present in khat, appears to only significantly inhibit CYP 1A2, CYP2A6, and CYP3A5 as compared to the other CYPs. These enzymes are among the most significantly expressed CYPs in the liver, with an expression rate of 10.3%, 3.8%, and 10.3% respectively (H. F. [75]). These CYPs are involved in the metabolism of various drugs including agomelatin, caffeine, clozapine, duloxetine, flutamide, lidocaine, olanzapine, propranolol, tacrine, tizanidine, zolmitriptan, melatonin, estradiol (for CYP1A2) [32], [65], artemisinin, coumarin, efavirenz, halothane, letrozole, nicotine, pilocarpine, tegafur and valproic acid (for CYP2A6) [65] and tacrolimus, and anticancer agents (e.g. sunitinib and vincristine)[65]. Besides that, CYP1A2 is also involved in the bioactivation of procarcinogens namely aromatic, heterocyclic amine, plycyclic aromatic hydrocarbons and aflatoxin B1 [32]. The inhibition of CYP1A2 would hinder metabolism and elimination of the aforementioned drugs/compounds. The fluoroquinolone antibiotic ciprofloxacin is a moderate potent inhibitor of CYP1A2 and was found to elevate plasma concentrations of tizanidine [30]. This interaction dangerously potentiates tizanidine’s hypotensive and sedative effects on patients by inhibiting CYP1A2 mediated metabolism when administered an hour earlier than tizanidine [30]. The inhibition of CYP2A6 for instance would hinder its role in bioactivation process of prodrug, tegafur, to 5′fluorouracil (5-FU) in cancer treatments [35]. However, inhibition of CYP2A6 by drugs or celery extracts may be beneficial as anti-smoking therapy because it reduces pro-carcinogen activation, biotransform nicotine into inactive cotinine besides activating tobacco-specific nitrosamines [11], [20]. CYP3A5 is structurally similar to CYP3A4 and therefore, shares substrate specificity [65]. CYP3A5 is responsible for metabolism of tacrolimus, and anticancer agents (e.g. sunitinib and vincristine) [65]. Post-transplant hypertension is common in organ transplant patients and therefore, concurrent use of antihypertensive drugs (e.g. dihydropyridine calcium channel blockers (CCBs)) with anti-rejection medications (e.g. tacrolimus) are common [72]. CCBs can inhibit CYP3A5 and prevent tacrolimus metabolism [72] leading to toxicity in high levels whereas organ rejection if concentrations are too low.

Earlier studies by Widler et al. showed that the maximal plasma concentration of cathinone was 127 ± 53 ng/ml) after 127 ± 30 min, with area under the plasma concentration – time curve (AUC) from 0 to 9 h of 415 ± 207 ng/ml per hour and terminal elimination half-life of 260 ± 102 min [69]. This study was in agreement with earlier study stating the absorption of khat leaves equivalent to 0.8 mg cathinone/kg body weight and cathinone oral dose of 0.5 mg/kg body weight [69]. A study by Halket et al. on the other hand observed that cathinone was hardly identified with approximately 20 ng/ml at 0.5 and 7.5 h after the start of khat chewing [34]. Peak plasma levels are achieved after 1.5–3.5 h of khat chewing with maximum levels ranging from 41 to 141 ng/ml (mean 83 ng/ml) per individual [34]. This suggested the slow release of khat alkaloids during chewing [34]. A study by Toennes et al. found that from 36.1 to 59.2 g of khat chewed, cathinone and cathine absorbed was 59 ± 21% and 84 ± 6% (mean ± SD) and C_max_ (maximal plasma concentration) of 58.9 ± 18.8 µg/L and 71.2 ± 13.9 µg/L respectively [64]. Khat sessions usually lasted over a period of 3 – 4 h with 100 – 300 g of khat leaves chewed. Our in vitro studies conveyed IC_50_ and K_i_ values ranging from 11.53 to 19.88 µM and 13.57 – 57.12 µM which were within 10 or 20-fold of C_max_, which indicates likelihood of drug interactions in spite of the milder risks.

Khat use have been found to hinder response to antipsychotic [33] and tuberculosis medication on patients [60]. Lipophilicity plays a major role in cellular uptake and ADMET (absorption, distribution, metabolism, excretion, toxicity) [68]. Besides dosage, the lipophilicity of cathinone should be known by healthcare professionals during administration of drugs to khat users. This is due to the statistically significant relationship between lipophilicity and drug and/or cathinone’s hepatic metabolism. Studies have suggested that high dose accompanied by an increase of lipophilicity is an unfavourable combination [16]. Khat compounds’ (e.g. cathinone) lipophilicity is important to be recognised as it may readily cross the blood brain barrier [5]. As compared to cathine, cathinone has more rapid onset of action which agrees to its higher lipophilicity nature facilitating its entry into the CNS but with a shorter duration of action which is in agreement with its rapid metabolism rate [54].

Further in vivo studies using animal models are warranted to investigate the effects of cathinone, cathine and other phytochemicals in khat plant on CYP gene expressions which is ongoing in our laboratory besides to validate the clinical relevance via human subjects to grasp effects of khat on all CYP isoforms including CYP1A2, CYP2A6, CYP2B6, CYP2C8, CYP2C19, CYP2E1, CYP2J2 and CYP3A5. Besides CYPs, there are other detoxification systems such as aldehyde oxidase (AOX), which is a non-CYP metabolic enzymes and cytosolic drug metabolising enzyme expressed in the human liver which is similar to CYPs which contributes to oxidation but act in the absence of NADPH cofactor [40], [63]. However, the in vivo importance of aldehyde oxidase remains unclear and thus, more efforts should be channelled to evaluate this aspect [63]. *In vivo* studies could assist the results by measuring the concentrations of main metabolite of a provided test drug in control and exposure groups. A decrease in the drug metabolites either from the test drug and khat exposure group could be used to distinguish whether khat causes an inhibition or otherwise, affecting the CYPs metabolism directly.

Despite the evidence that cathinone inhibited a few important drug metabolising CYPs in vitro, the in vivo processes can be entirely divergent. The limitations of this study includes; (1) fluorescent substrates may interact with CYP differently than classic drug substrates which is why they are used as initial screening tools only; (2) recombinant CYP is very simple compared to microsomes or hepatocytes; and (3) CYP inhibition must be confirmed using more relevant drug substrates with in vitro (e.g. liver microsomes, hepatocytes); (4) in vivo clinical studies. Moreover, in vitro study could not encompass pharmacokinetic factors such as gastric digestion, absorption of cathinone and first-pass effect in human especially if cathinone is administered orally, the cathinone concentration will be greatly reduced before reaching the systemic circulation. Worth mentioning is that the present study uses only one substrate for each CYP isoform which could be improved by using different substrates to compare the possible interactions. Cathinone is often blamed for the dependence and/or addiction issue ascribed to khat chewing [25]. However, besides cathinone and cathine, khat also contains other alkaloids like cathedulins, whose pharmacologic effects remain mostly unknown [25]. Khat plant touches the living of millions of people in the khat-belt countries and therefore, the research community ought to put forward means to maximise the value while minimising the negative impacts of khat use. In addition, future studies are recommended to evaluate effects of cathinone on the CYP1A family as they are present in high amount in the enteric cells as the gut is the second absorption area during khat intake. *In silico* studies on interaction of other khat constituents for instance cathine, tannins, cathedulins, just to name a few, with CYPs are warranted to visualise the possible interactions that is worthy to be explored.

## 5 Conclusion

This study is an initial screening that must be confirmed using more accurate models. Cathinone inhibited CYP1A2, CYP2A6 and CYP3A5 only but showed negligible in vitro inhibitory effects on CYP2B6, CYP2C8, CYP2C19, CYP2E1 and CYP2J2. CYP1A2, CYP2A6 and CYP3A5 are among the major human drug metabolising enzymes, responsible for metabolising various commonly used medications including antibiotics, immunosuppressive drugs, anticoagulants, anticancer drugs as well as painkillers. Cathinone inhibited CYP1A2, CYP2A6 and CYP3A5 via non-competitve and uncompetitive mixed mode. Docking outcome demonstrated the competitive inhibition of cathinone on CYPs were similar to prior research utilizing CYPs’ inhibitors, that binds closely to the haem group and surrounding residues. Conserved amino acid residues were also involved in cathinone-CYPs binding. In addition, hydrophobic, hydrogen bond and π-stacking interactions are among the type of interactions contributing to inhibitory activity of cathinone on CYP1A2, CYP2A6 and CYP3A5. Our study demonstrates that khat consumption may result in inhibition of CYP1A2, CYP2A6, and CYP3A5 through its major active component, cathinone, with potential clinically-significant implications for users who may be taking prescribed medications that are metabolised through these pathways. It would therefore be prudent for healthcare professionals to be aware of these potential interactions during their prescribing and treatment of khat users, either by advising abstinence or adjusting their prescribing accordingly.

## CRediT authorship contribution statement

**Sharoen Yu Ming Lim:** Conceptualization, Formal analysis, Investigation, Data curation, Writing – original draft, Writing – review & editing, Visualization, Project administration. **Jason Siau Ee Loo:** Software, Formal analysis, Writing – review & editing, Visualization, Supervision. **Mustafa Alshagga:** Conceptualization, Methodology, Resources, Supervision, Funding acquisition **Mohammed Abdullah Alshawsh:** Conceptualization, Resources. **Chin Eng Ong:** Conceptualization, Methodology **Yan Pan:** Conceptualization, Methodology, Validation, Resources, Writing – review & editing, Supervision.

## Funding

This work was supported by the University of Nottingham Malaysia [grant number 54-ER-0-744019].

## Conflict of interest

None.

## Declaration of Competing Interest

The authors declare that they have no known competing financial interests or personal relationships that could have appeared to influence the work reported in this paper.
