# Supplementary material for: In vitro and In silico studies of interactions of cathinone with human recombinant cytochrome P450 CYP(1A2), CYP2A6, CYP2B6, CYP2C8, CYP2C19, CYP2E1, CYP2J2, and CYP3A5
Source: Toxicol Rep. 2022 Mar 30;9:759–68. doi: 10.1016/j.toxrep.2022.03.040 (PMC9742833; doi:10.1016/j.toxrep.2022.03.040)
Supplement: Supplementary file 1 — Supplementary material [file mmc1.docx]

**Supplementary Information**

**Time Dependent Inhibition**

**Figure 1.** Time dependent inhibition of cathinone on (A) CYP1A2, (B) CYP2A6, (C) CYP2B6, (D) CYP2C8, (E) CYP2C19, (F) CYP2E1, (G) CYP2J2 and (H) CYP3A5. IC_50_ values were determined by non-linear regression analysis using GraphPad Prism version 9 for Windows (GraphPad Software, La Jolla California, USA). Each point represents mean ± SD (n=3). *CYP* Cytochrome P450, *IC_50_* 50% inhibitory concentration

Lineweaver-Burk’s plot

C

**Figure 2.** The Lineweaver-Burk plot or double reciprocal was plotted with inverse velocity (1/V) against the inverse of the substrate concentrations (1/[S]). Lineweaver-Burk plot (A) of inhibition of CYP1A2 by Cathinone; (B) of inhibition of CYP2A6 by Cathinone and (C) of inhibition of CYP3A5 by Cathinone at the indicated concentrations of Cathinone and substrate. The substrate concentration used were; EOMCC (1.5, 3, 6, 12 µM) for CYP1A2, CC (5, 10, 20, 40µM) for CYP2A6 and BOMCC (5, 10, 20, 40 µM) for CYP3A5. The secondary plots were plotted using slopes from Lineweaver-burk plot against cathinone concentrations which was used to derive the K_i_, inhibition constant values. Each data point are triplicates that was represented by mean ± SD (n=3).
